# Dysidinoid A, an Unusual Meroterpenoid with Anti-MRSA Activity from the South China Sea Sponge *Dysidea* sp.

**DOI:** 10.3390/molecules191118025

**Published:** 2014-11-05

**Authors:** Wei-Hua Jiao, Jing Li, Qian Liu, Ting-Ting Xu, Guo-Hua Shi, Hao-Bing Yu, Fan Yang, Bing-Nan Han, Min Li, Hou-Wen Lin

**Affiliations:** 1 Key Laboratory for Marine Drugs, Department of Pharmacy, Renji Hospital, School of Medicine, Shanghai Jiaotong University, Shanghai 200127, China; E-Mails: weihuajiao@hotmail.com (W.-H.J.); 120571114@163.com (T.-T.X); shigh88804@hotmail.com (G.-H.S.); bill1985@126.com (F.Y.); bingnanh@hotmail.com (B.-N.H.); 2 College of Food and Biological Engineering, Jimei University, Xiamen 361021, China; E-Mail: lijingjmu@163.com; 3 Department of Clinical Laboratory, Renji Hospital, School of Medicine, Shanghai Jiaotong University, Shanghai 200127, China; E-Mails: qq2005011@163.com (Q.L.); ruth_limin@126.com (M.L.); 4 Laboratory of Marine Drugs, Department of Pharmacy, Changzheng Hospital, Second Military Medical University, Shanghai 200003, China; E-Mail: yuhaobing1986@126.com

**Keywords:** dysidinoid A, meroterpenoid, anti-MRSA, *Dysidea*, marine sponge

## Abstract

An unusual meroterpenoid, dysidinoid A (**1**), was isolated from the South China Sea sponge *Dysidea* sp. Its structure was elucidated by extensive spectroscopic methods including HRESIMS and 2D NMR, and its absolute configuration was determined by single-crystal X-ray diffraction analysis. Dysidinoid A (**1**) is the first meroterpenoid from Nature bearing a 9,4-friedodrime skeleton and a 2,5-dionepyrrole unit. Dysidinoid A (**1**) showed potent antibacterial activity against two strains of pathogenic bacteria methicillin-resistant *Staphylococcus aureus* (MRSA) with MIC_90_ values of 8.0 μg/mL against both.

## 1. Introduction

Infectious diseases are the leading cause of death worldwide. Emerging infections due to methicillin resistant *Staphylococcus aureus* (MRSA) pose a significant threat to patients [1,2]. It has been estimated that in the United States more people die from MRSA related infections than from HIV [3]. Infections involving drug resistant bacteria are more difficult to treat due to increased costs and decreased efficacies [4,5]. One important approach to drug discovery for the treatment of MRSA is through natural products research.

Marine sponges of the genus *Dysidea* (order Dictyoceratida, family Dydideidae) have proven to be prolific producers of structurally diverse secondary metabolites, such as sesequiterpene quinones [6,7,8], sesquiterpenoids [9], diterpenoids [10], sterols [11], and polychlorinated compounds [12,13,14]. These metabolites showed a spectrum of interesting biological activities, including antifungal [15], antibacterial [16], antitumor [17,18], anti-inflammatory [15,19], and antioxidative activities [20].

**Figure 1:**
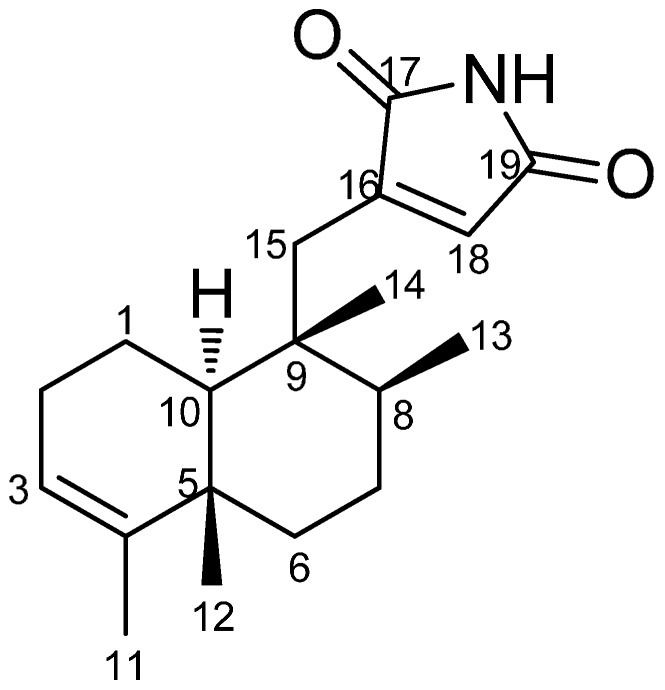
The chemical structure of dysidinoid A (**1**).

In our efforts to search for new anti-MRSA agents from marine sponges collected from the South China Sea, chemical investigation of an active fraction from the sponge *Dysidea* sp. resulted in the isolation of a novel meroterpenoid, dysidinoid A (**1**) (Figure 1). It is the first meroterpenoid from Nature bearing a 9,4-friedodrime skeleton and a 2,5-dionepyrrole. Antibacterial evaluation showed that dysidinoid A showed potent antibacterial activity against two strains of pathogenic bacteria MRSA with MIC_90_ values of 8.0 μg/mL against both. Details of structural elucidation and antibacterial activity of dysidinoid A (**1**) were reported herein.

## 2. Results and Discussion

Dysidinoid A (**1**) was obtained as colorless needles with 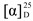 +35.4 (*c* 0.50, MeOH). Its IR spectrum showed absorption bands assignable to amide (3276 cm^−1^) and carbonyl (1775 and 1714) functionalities. The positive ESIMS of **1** exhibited quasimolecular ion peaks at *m*/*z* 302.2 [M+H]^+^ and 324.2 [M+Na]^+^, respectively. The molecular formula of C_19_H_27_NO_2_ with seven degrees of unsaturation, was deduced from HRESIMS at *m*/*z* 324.1941 [M+Na]^+^ (calcd. for C_19_H_27_NO_2_, 324.1939), which was supported by the ^1^H- and ^13^C-NMR data (Table 1). The ^1^H-NMR spectrum of **1** showed resonances attributable to two olefinic protons at δ_H_ 5.16 (H-3) and 6.26 (H-18), three tertiary methyl groups at δ_H_ 1.55 (H_3_-11), 1.00 (H_3_-12), and 0.88 (H_3_-14), a secondary methyl group at δ_H_ 0.95 (H_3_-13). In addition, the spectrum showed resonances due to an exchangable amine proton at δ_H_ 7.33 (20-NH), as well as partially overlapping signals with complex coupling patterns between δ_H_ 1.08 and 2.61 that could be attributed to several aliphatic methylene and methine units. The ^13^C-NMR and DEPT spectra of **1** showed 19 carbon resonances, corresponding to two carbonyl groups (δ_C_ 171.7 and 170.4), two olefinic quaternary carbons (δ_C_ 143.9 and 147.9), two aliphatic quaternary carbons (δ_C_ 38.3 and 42.4), two olefinic methine carbons (δ_C_ 120.5 and 130.4), two aliphatic methine carbons (δ_C_ 37.4 and 47.0), five aliphatic methylene carbons (δ_C_ 19.0, 26.3, 36.2, 27.4, and 32.5), and four methyl carbons (δ_C_ 17.7, 19.8, 16.3, and 18.0). The above spectroscopic signatures suggested the presence of a 9,4-friedodrime sesquiterpene moiety and accounted for four degrees of unsaturation, indicating three rings in the structure of **1**.

**Table 1:** The ^1^H- (600 MHz) and ^13^C- (150 MHz) NMR data of compound **1** in CDCl_3_. ^a^

Position	δ_C_	δ_H_ (*J* in Hz)	HMBC (H→C)	NOESY
1α	19.0, CH_2_	1.83, m	C-2, 3, 5, 9, 10	H-1β, 2β, 10
1β		1.53, m	C-2, 5, 10	H_3_-12, 14, H-1α, 2β
2α	26.3, CH_2_	1.93, m		H-1α, 1β, 10
2β		2.07, m	C-3, 4, 10	H-1α, 1β, 2α
3	120.5, CH	5.16, br s	C-5, 11	H_3_-11, H-2α, 2β
4	143.9, C			
5	38.3, C			
6α	36.2, CH_2_	1.08, m	C-8	H-6β, 7a, 8, 10
6β		1.68, dt (12.8, 3.4)	C-7, 8, 10, 12	H_3_-11, 12, H-6α, 7b
7a	27.4, CH_2_	1.41, m	C-5, 6, 9, 13	H-6, 8
7b		1.40, dd (6.9, 3.5)		H-6, 8, H_3_-12, 13, 14
8	37.4, CH	1.28, m	C-7, 9, 13	H-7b, 10, H_3_-13
9	42.4, C			
10	47.0, CH	1.12, dd (12.4, 1.6)	C-2, 4, 5, 9, 12, 14, 15	H-1α, 2α, 8, 15α, 15β
11	17.7, CH_3_	1.55, br s	C-3, 4, 5	H_3_-12, H-3
12	19.8, CH_3_	1.00, s	C-4, 5, 6, 10	H_3_-11, 14, H-6β, 7β
13	16.3, CH_3_	0.95, d (6.7)	C-7, 8, 9	H_3_-14, H-7β, 8
14	18.0, CH_3_	0.88, s	C-8, 9, 10, 15	H_3_-12, 13, H-1β, 7β
15α	32.5, CH_2_	2.61, d (14.1)	C-8, 9, 10, 14, 16, 17, 18	H_3_-14, H-1α, 10
15β		2.43, dd (14.1, 1.2)	C-8, 9, 10, 14, 16, 17, 18	H_3_-13
16	147.9, C			
17	171.7, C			
18	130.4, CH	6.26, d (1.0)	C-15, 16, 19	H_3_-13, H-10, 15α, 15β
19	170.4, C			
20-NH		7.33, br s		

^a^ Assignments of the ^13^C and ^1^H signals were made on the basis of HSQC spectroscopic data.

**Figure 2:**
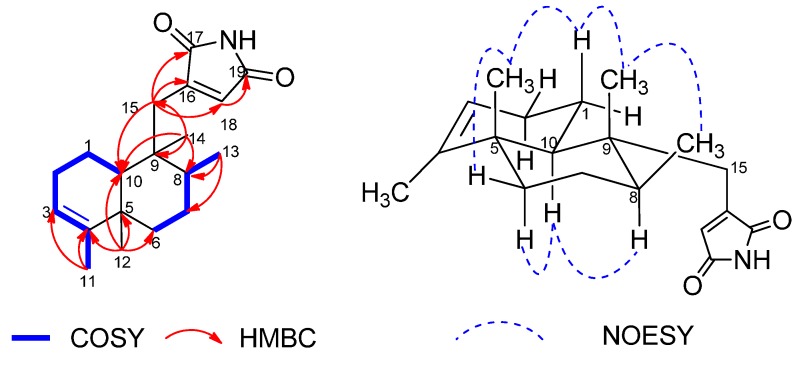
Key COSY, HMBC, and NOESY correlations of dysidinoid A (**1**).

Unambiguous assignment of NMR data of **1** was achieved by a combination of COSY, HSQC, and HMBC experiments, as depicted in Figure 2. In the ^1^H-^1^H COSY spectrum, the correlations of H_2_-1/H_2_-2/H-3, H_2_-6/H_2_-7/H-8/H_3_-13, and allylic coupling correlations of H-3/H_3_-11 revealed the presence of two fragments (thick lines in Figure 2). The two spin systems and their connectivity with the remaining atoms enabled assembly into the final planar structure based upon the HMBC spectrum of **1**. The HMBC correlations from H_3_-11 to C-3, C-4, and C-5, from H_3_-12 to C-4, C-5, C-6 and C-10, from H_3_-13 to C-7, C-8, and C-9, and H_3_-14 to C-8, C-9, C-10, and C-15 indicated the presence of 9,4-friedodrime sesquiterpene skeleton with four methyl groups at C-4, C-5, C-8, and C-9, respectively. This assignment was confirmed by the HMBC correlations from H-10 to C-2, C-4, C-5, C-9, C-12, C-14, and C-15. Furthermore, the olefinic proton H-18 showed HMBC correlations with C-15, C-16, and C-19, in combination with the chemical shifts of the proton and carbon resonances, suggested the presence of a 2,5-dionepyrrole substructure. In addition, HMBC correlations from the methylene protons H_2_-15 to C-8, C-9, C-10, C-14, C-16, C-17, and C-18 supported the linkage of C-9 and C-16 via the methylene CH_2_-15 between the 9,4-friedodrime sesquiterpene moiety and 2,5-dionepyrrole substructure. Therefore, the gross structure of **1** was determined as shown in Figure 2.

The relative configuration of **1** was deduced from NOESY correlations in combination with coupling constant values. The large coupling constant between H-1β and H-10 (*J* = 12.4 Hz) and the NOESY correlations of H-1β/H_3_-12 and H_3_-14 indicated the axial orientations of these protons and methyls and also revealed the *trans* fusion of the two six-numbered rings [15,18]. The NOESY correlation of H_3_-13/H_3_-14 and H_3_-12/H_3_-14 revealed the three methyl groups are all β-orientation, while NOESY correlations from H-8 to H-6α, and H-10 suggested the three protons were α-orientation.

**Figure 3:**
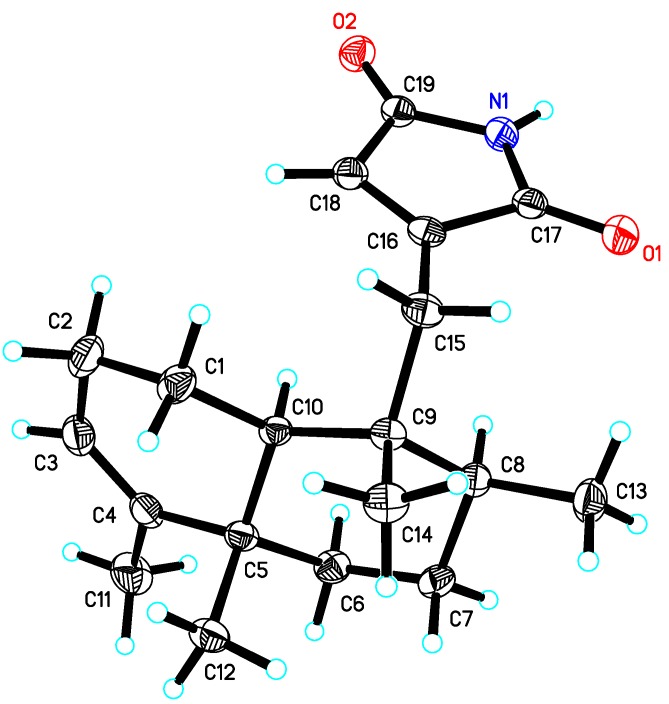
X-ray ORTEP drawing of dysidinoid A (**1**).

Fortunately, crystals of **1** suitable for single crystal X-ray diffraction analysis were obtained from a methanol solution. The relative configuration of **1** was unambiguously established by its X-ray crystal structure (Figure 3). Besides, a final refinement of the CuKa diffraction data resulted in the assignment of the absolute configuration of **1** as 5*S*, 8*S*, 9*R*, and 10*S*.

Minimal inhibitory concentration (MIC) was detected to evaluate the antimicrobial activities of dysidinoid A (**1**) toward two strains of hospital-acquired methicillin-resitant *Staphylococcus aureus* (MRSA H0556 and MRSAH0117). Dysidinoid A (**1**) showed potent inhibitory activity against MRSA with MIC_90_ values of 8 μg/mL, and chloromycetin was used as positive control (MIC_90_ 2 μg/mL), while methicillin was used as negative control (MIC_90_ 128 μg/mL).

## 3. Experimental Section

### 3.1. General Experimental Procedures

Optical rotations were recorded on an Autopol I polarimeter (No. 30575, Rudolph Research Analytical, Perkin-Elmer, Inc., Waltham, MA, USA) with a 10 cm length cell at room temperature. UV and IR (KBr) spectra were recorded on a Hitachi U-3010 spectrophotometer (Hitachi, Inc., Tokyo, Japan) and Jasco FTIR-400 spectrometer (Jasco Inc., Tokyo, Japan), respectively. ^1^H, ^13^C, DEPT135, COSY, HSQC, HMBC, and NOESY NMR spectra were recorded at room temperature on a Bruker Avance DRX-600 MHz NMR spectrometer ((Bruker Biospin Corp., Billerica, MA, USA) with CDCl_3_ as the solvent. HRESIMS spectra were measured on an Agilent 6210 LC/MSD TOF mass spectrometer (Agilent, Milford, MA, USA). Column chromatography was conducted using pre-coated silica gel (65 × 250 or 230 × 400 mesh). Sephadex LH-20 was purchased from Amersham Pharmacia Biotech AB (Pharmacia Fine Chemicals, Piscataway, NJ, USA). Purification of the compounds was performed using a Waters Alliance 2695 separation module equipped with a Waters 2998 Photodiode Array (PDA) detector (Waters Corp., Milford, MA, USA).

### 3.2. Animal Material

Samples of *Dysidea* sp. were collected along the coast of Yongxing Island in Xiasha on 12 April 2010. The voucher number for this collection is XD10401, and a voucher sample is maintained at the Key Laboratory for Marine Drugs, Department of Pharmacy, Renji Hospital, School of Medicine, Shanghai Jiaotong University, Shanghai, China. The sponge was identified by Professor Jin-He Li (Institute of Oceanology, Chinese Academy of Science).

### 3.3. Extraction, Isolation and Characterization

The animals (200 g, dry weight) were soaked in EtOH (250 mL, 25 °C, 72 h) repeatedly to give 24.6 g of a crude EtOH extract after solvent removal. The extract was dissolved in 250 mL H_2_O, and partitioned five times with the same volume of CH_2_Cl_2_ to yield after concentration 12.0 g of a CH_2_Cl_2_ solvent extract, The CH_2_Cl_2_-soluble fraction was subjected silica gel column chromatography eluting with a gradient of CH_2_Cl_2_ and MeOH, yielding four subfractions (D1–D4). Fraction D3 (1.4 g) was passed through an ODS chromatography column eluted with a gradient of aqueous MeOH, size-exclusion chromatography Sephadex LH-20 eluted with CH_2_Cl_2_/MeOH (1:1), and then purified by reversed-phase HPLC (YMC-Park Pro C18, 10 mm × 250 mm, 2 mL/min, 280 nm) with 65% CH_3_CN, to give dysidinoid A (**1**, 4.3 mg, *t*_R_ 26.5 min); colorless needles (MeOH); 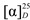 +35.4 (*c* 0.5, MeOH); UV (MeOH) λ_max_ (log ε) 209 (4.05), 235 (398) nm; ^1^H and ^13^C-NMR, see Table 1; IR (KBr) υ_max_ 3276, 2961, 2928, 2857, 1775, 1714, 1621, 1453, 1344, 1124, 1075, 871, 626 cm^−1^; positive ESIMS *m*/*z* 302.2 [M+H]^+^, 324.2 [M+Na]^+^; positive HRESIMS *m*/*z* 324.1941 [M+Na]^+^ (calcd for C_19_H_27_NO_2_, 324.1939).

### 3.4. X-ray Crystallographic Analysis of Dysidinoid A (**1**)

C_19_H_27_NO_2_, colorless blocks, *M* = 301.42, Orthorhombic, *P*2_1_, *a* = 7.4098(2) Å, *b* = 14.0638(3) Å, *c* = 16.2014(3) Å, α = β = γ = 90°, *V* = 1688.35(7) Å^3^, *Z* = 4, *D_x_* = 1.186 mg/m^3^, *F* (000) = 656, μ(Cu-Kα) = 0.594 mm^−1^, crystal dimensions 0.30 × 0.16 × 0.10 mm^3^ were used for measurement on a SMART CCD using graphite monochromated radiation (λ = 1.54178 Å); 5416 unique reflections were collected to θ_max_ = 69.73°. The structure was solved by direct methods (Shelxs97) and refined by full-matrix least-squares on *F*^2^. Hydrogen atoms were located by the geometric calculation method and difference Fourier method. The final *R*_1_ = 0.0358, *wR*_2_ = 0.1083 (w = 1/σ|F|^2^) and *S* = 1.012. Crystallographic data for dysidinoid A (**1**) have been deposited with the Cambridge Crystallographic Data Center as supplementary publication No. CCDC 1029972. Copies of the data can be obtained, free of charge, on application to the Director, CCDC, 12 Union Road, Cambridge CB2 1EZ, UK (Fax: +44-(0)1223-336033, or E-Mail: deposit@ccdc.cam.ac.uk).

### 3.5. Antimicrobial Assays

Minimum inhibitory concentration (MIC) was determined according to Clinical and Laboratory Standards Institute (CLSI) guidelines. The MIC_90_ values were recorded using a spectrophotometer. For antibiotic sensitivity assays, bacteria in 96-well plates (Corning) were incubated with dysidinoid A (**1**) or antibiotic standards at final concentrations of 0 to 256 mg/mL. The plates were incubated at 37 °C and read at 24 h.

## 4. Conclusions

Marine sponges provide a rich source on drug discovery for the treatment of MRSA infectious diseases. In this paper, dysidinoid A (**1**), an unusual meroterpenoid, was isolated from the South China Sea sponge *Dysidea* sp. Its structure was determined based on extensive spectroscopic data, and the absolute configuration of **1** was established by single-crystal X-ray diffraction analysis. Dysidinoid A (**1**) showed potent antibacterial activity against two strains of hospital-acquired pathogenic MRSA with MIC_90_ values of 8.0 μg/mL against both.

## Supplementary Materials

Supplementary materials can be accessed at: http://www.mdpi.com/1420-3049/19/11/18025/s1.

## Supplementary Files

Supplementary File 1
