# Supplementary material for: Dysidinoid A, an Unusual Meroterpenoid with Anti-MRSA Activity from the South China Sea Sponge Dysidea sp
Source: Molecules. 2014 Nov 5;19(11):18025–32. doi: 10.3390/molecules191118025 (PMC6270960; doi:10.3390/molecules191118025)
Supplement: Supplementary File 1 [file molecules-19-18025-s001.pdf]

Supplementary Materials

Contents

Figure S1. ^{1}H-NMR Spectrum of Dysidinoid A (**1**) in $CDCl_3$.
Figure S2. ^{13}C-NMR Spectrum of Dysidinoid A (**1**) in $CDCl_3$.
Figure S3. DEPT135 Spectrum of Dysidinoid A (**1**) in $CDCl_3$.
Figure S4. ^{1}H-^{1}H COSY Spectrum of Dysidinoid A (**1**) in $CDCl_3$.
Figure S5. HSQC Spectrum of Dysidinoid A (**1**) in $CDCl_3$.
Figure S6. HMBC Spectrum of Dysidinoid A (**1**) in $CDCl_3$.
Figure S7. NOESY Spectrum of Dysidinoid A (**1**) in $CDCl_3$.
Figure S8. HRESIMS of Dysidinoid A (**1**).
Figure S9. UV spectrum of Dysidinoid A (**1**) in MeOH.
Figure S10. IR spectrum of Dysidinoid A (**1**).

Figure S1. ^{1}H-NMR Spectrum of Dysidinoid A (**1**) in $CDCl_3$.

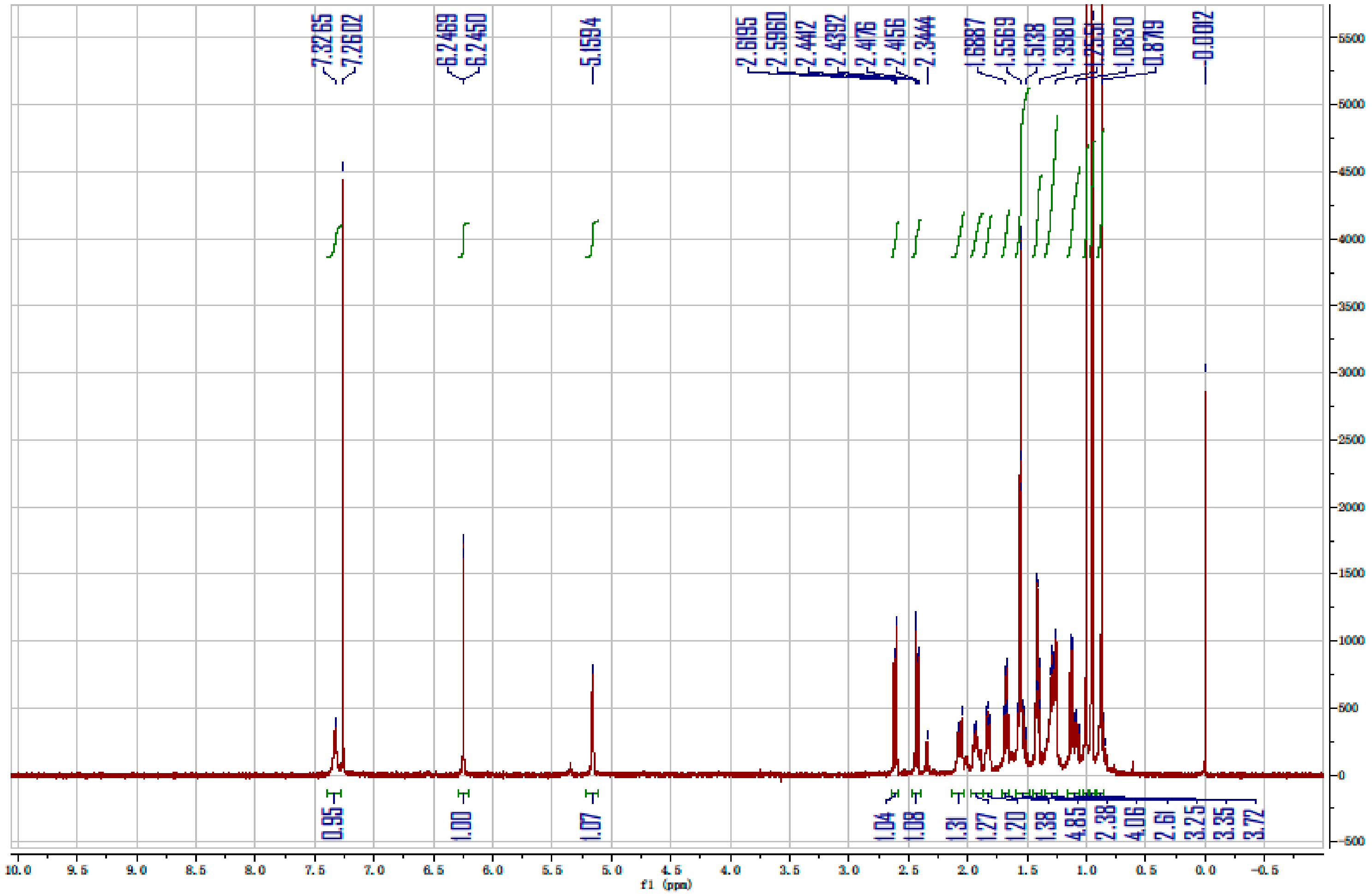

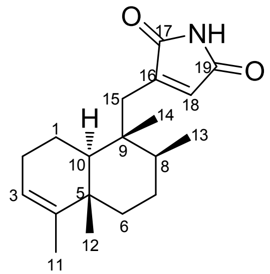

Dysidinoid A (**1**)

Figure S2. ^{13}C-NMR Spectrum of Dysidinoid A (**1**) in $CDCl_3$.

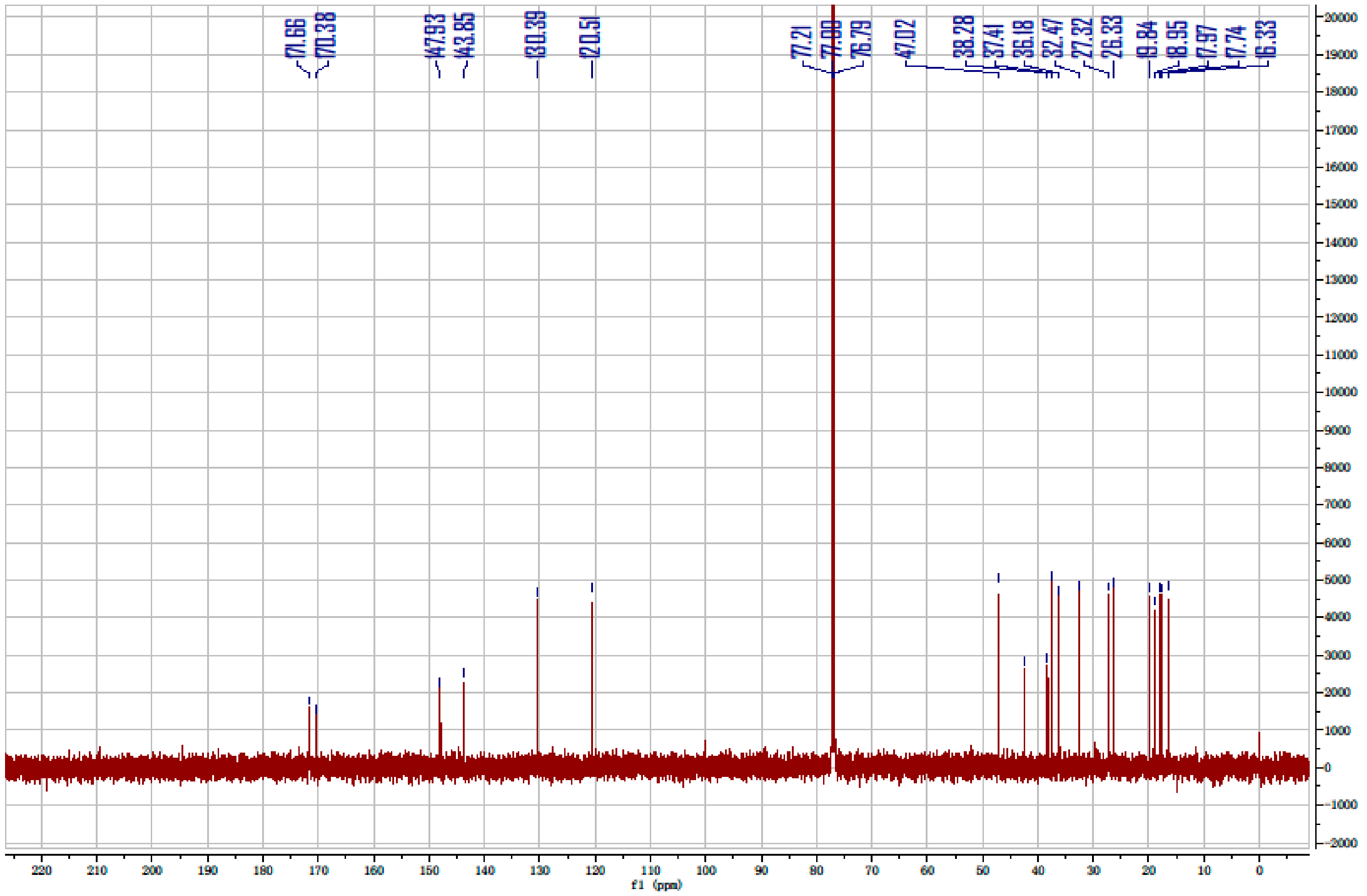

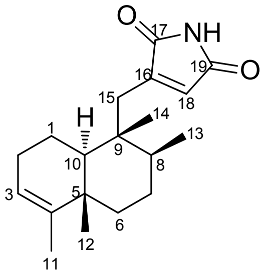
Dysidinoid A (**1**)

Figure S3. DEPT135 Spectrum of Dysidinoid A (**1**) in $CDCl_3$.

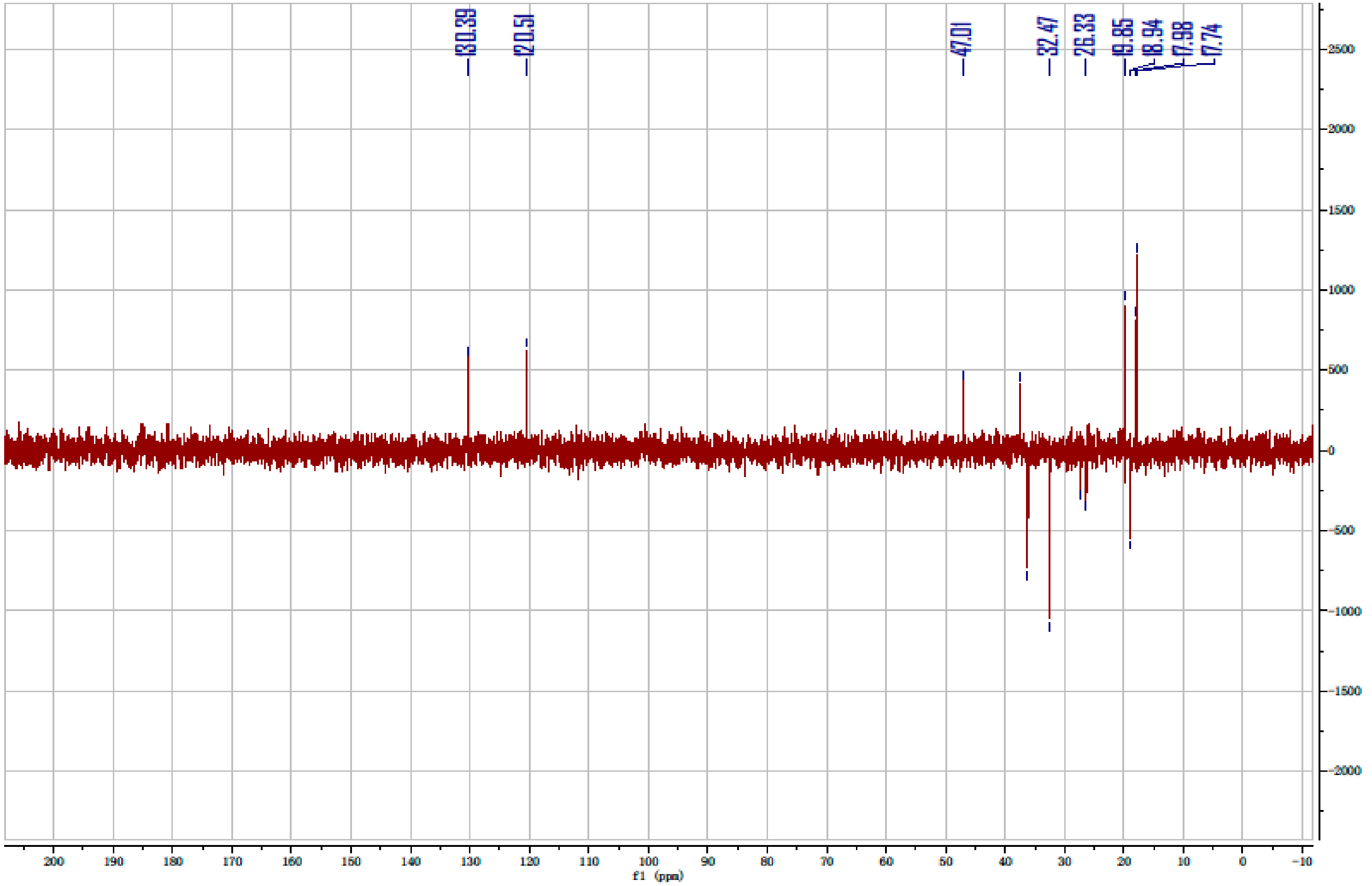

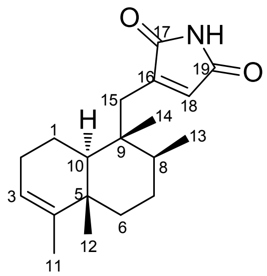

Dysidinoid A (**1**)

Figure S4. ^{1}H-^{1}H COSY Spectrum of Dysidinoid A (**1**) in $CDCl_3$.

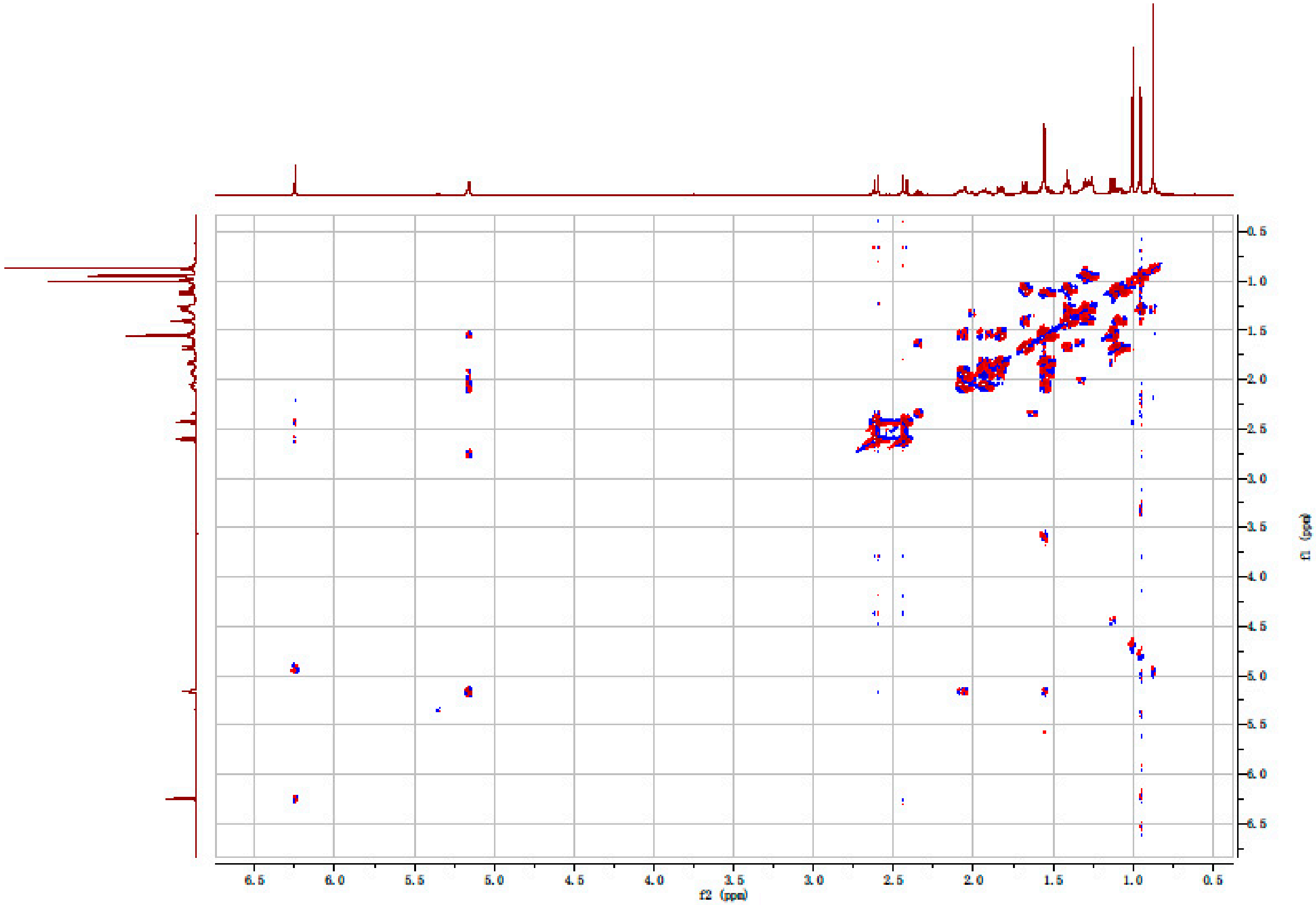

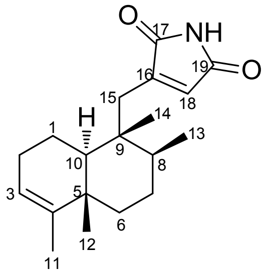

Dysidinoid A (**1**)

Figure S5. HSQC Spectrum of Dysidinoid A (**1**) in $CDCl_3$.

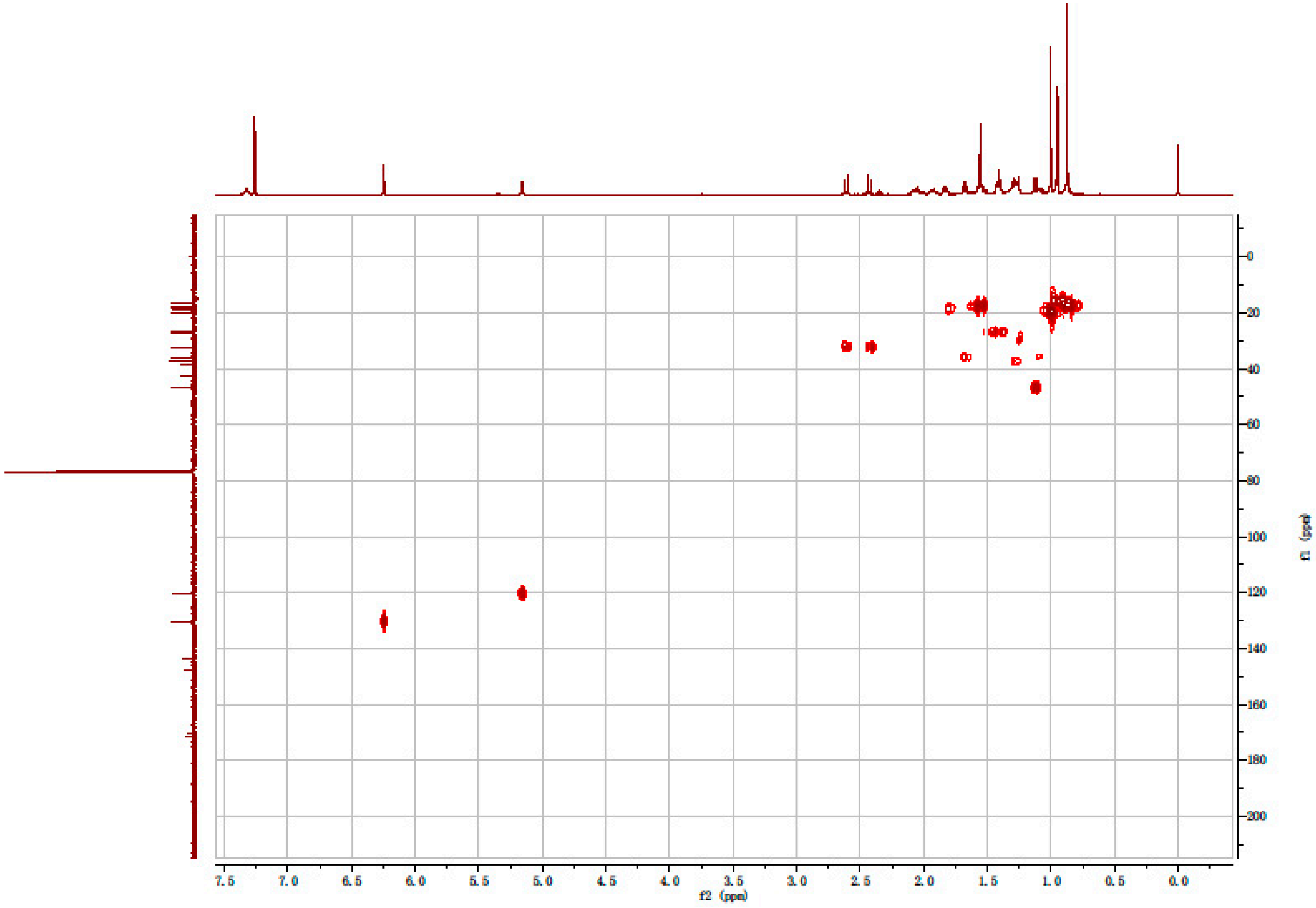

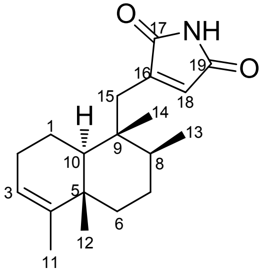

Dysidinoid A (**1**)

Figure S6. HMBC Spectrum of Dysidinoid A (**1**) in CDCl3.

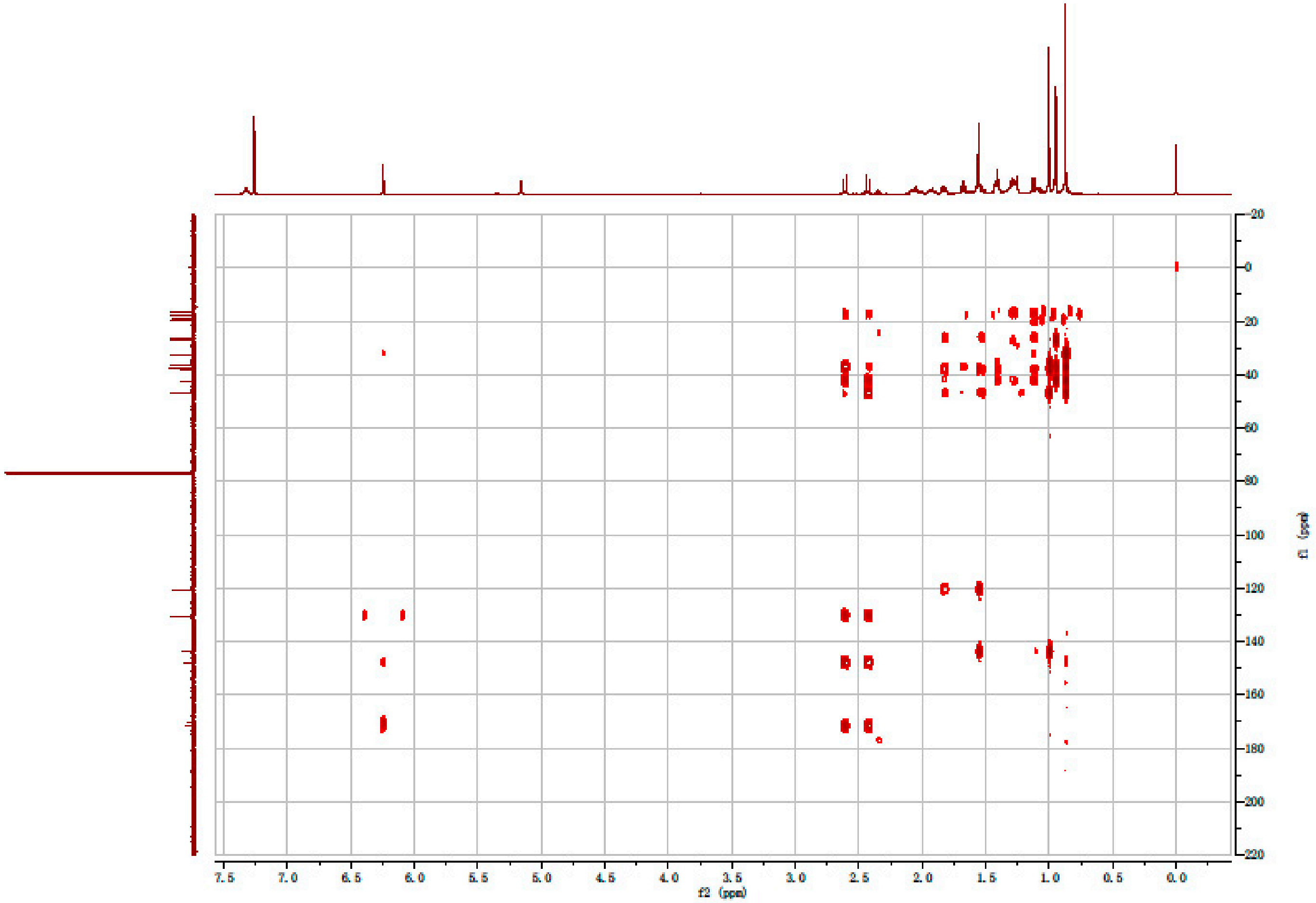

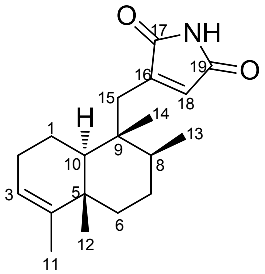

Dysidinoid A (**1**)

Figure S7. NOESY Spectrum of Dysidinoid A (**1**) in $CDCl_3$.

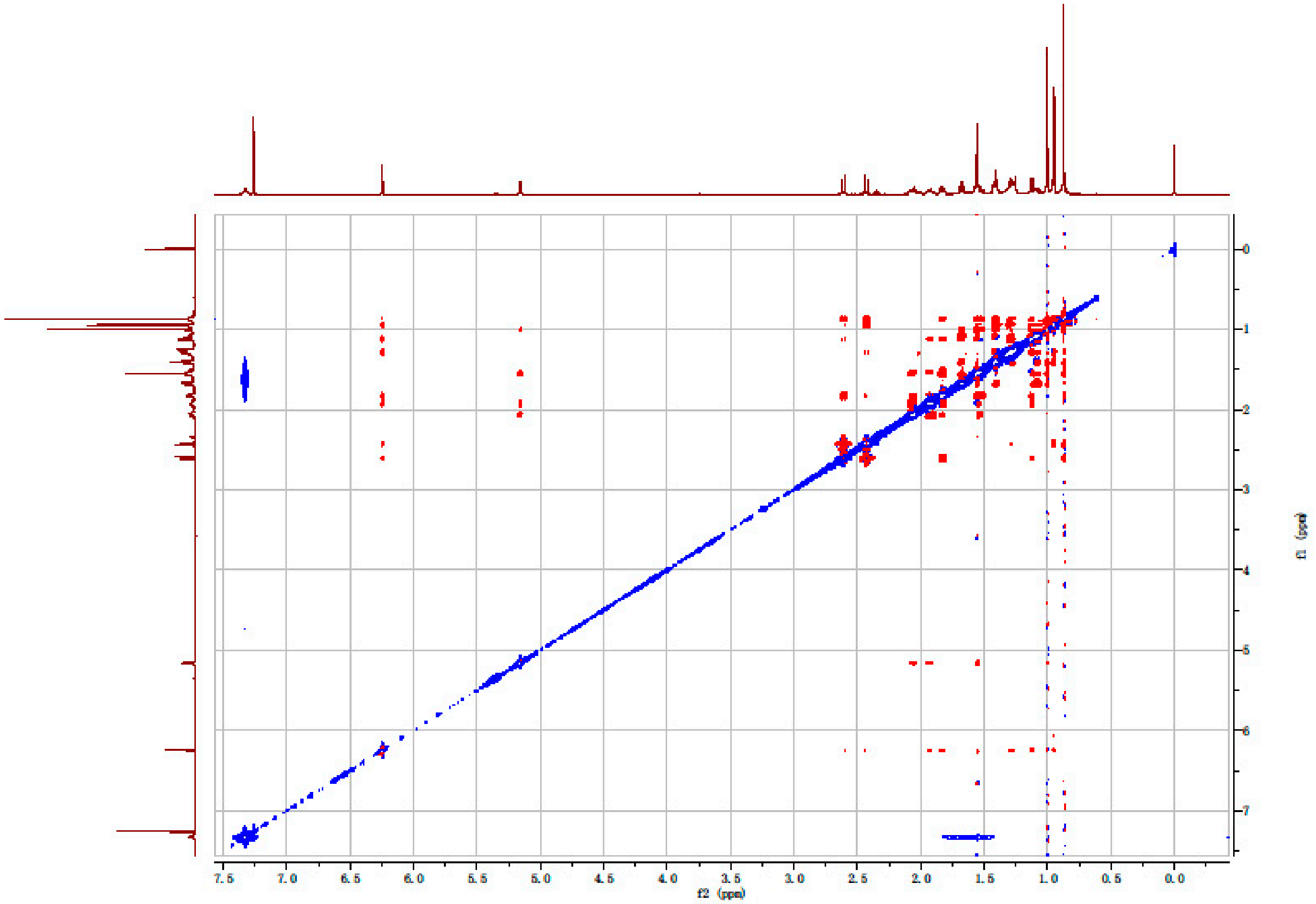

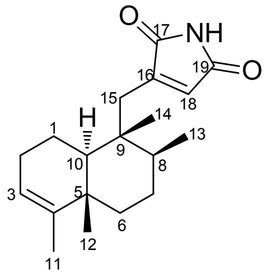

Dysidinoid A (**1**)

Figure S8. HRESIMS of Dysidinoid A (**1**).

Elemental Composition Report **Page 1**

Tolerance = 10.0 PPM / DBE: min = -1.5, max = 50.0
Selected filters: None

Monoisotopic Mass, Even Electron Ions
2 formula(e) evaluated with 1 results within limits (up to 50 closest results for each mass)
Elements Used:
C: 5-20 H: 5-30 N: 1-1 O: 1-2 Na: 1-1

SIPI
PI0203 M.W=301
WQ12-471H1 44 (1.522) AM (Cen,4, 80.00, Ar,5000.0,337.18,0.70); Sm (SG, 2x1.00); Cm (42:54)

Q-Tof micro
YA019

19-Nov-2012,11:15:55

TOF MS ES+
9.51e3

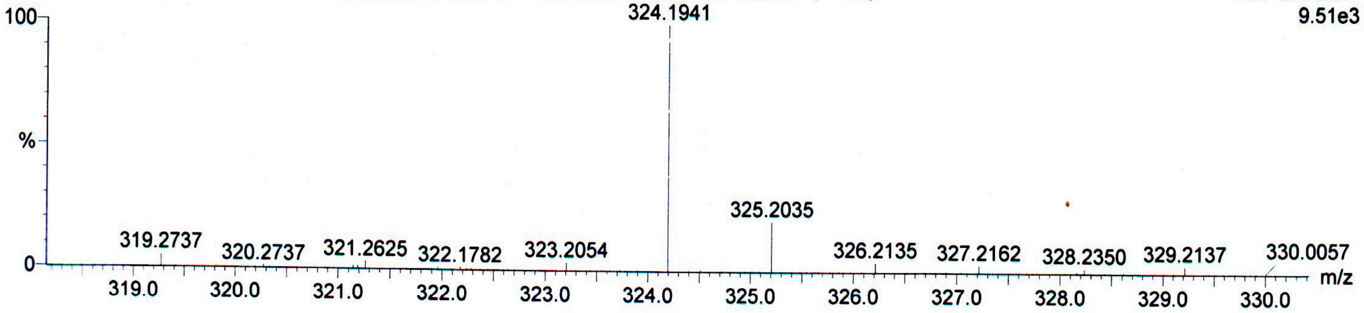

Minimum:	71.00				-1.5		
Maximum:	100.00		5.0	10.0	50.0		
Mass	RA	Calc. Mass	mDa	PPM	DBE	i-FIT	Formula
324.1941	100.00	324.1939	0.2	0.6	6.5	32.4	C19 H27 N O2 Na

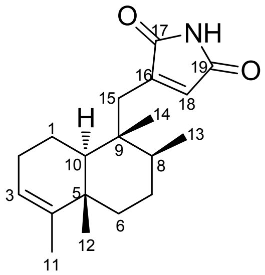

Dysidinoid A (**1**)

Figure S9. UV spectrum of Dysidinoid A (**1**) in MeOH.

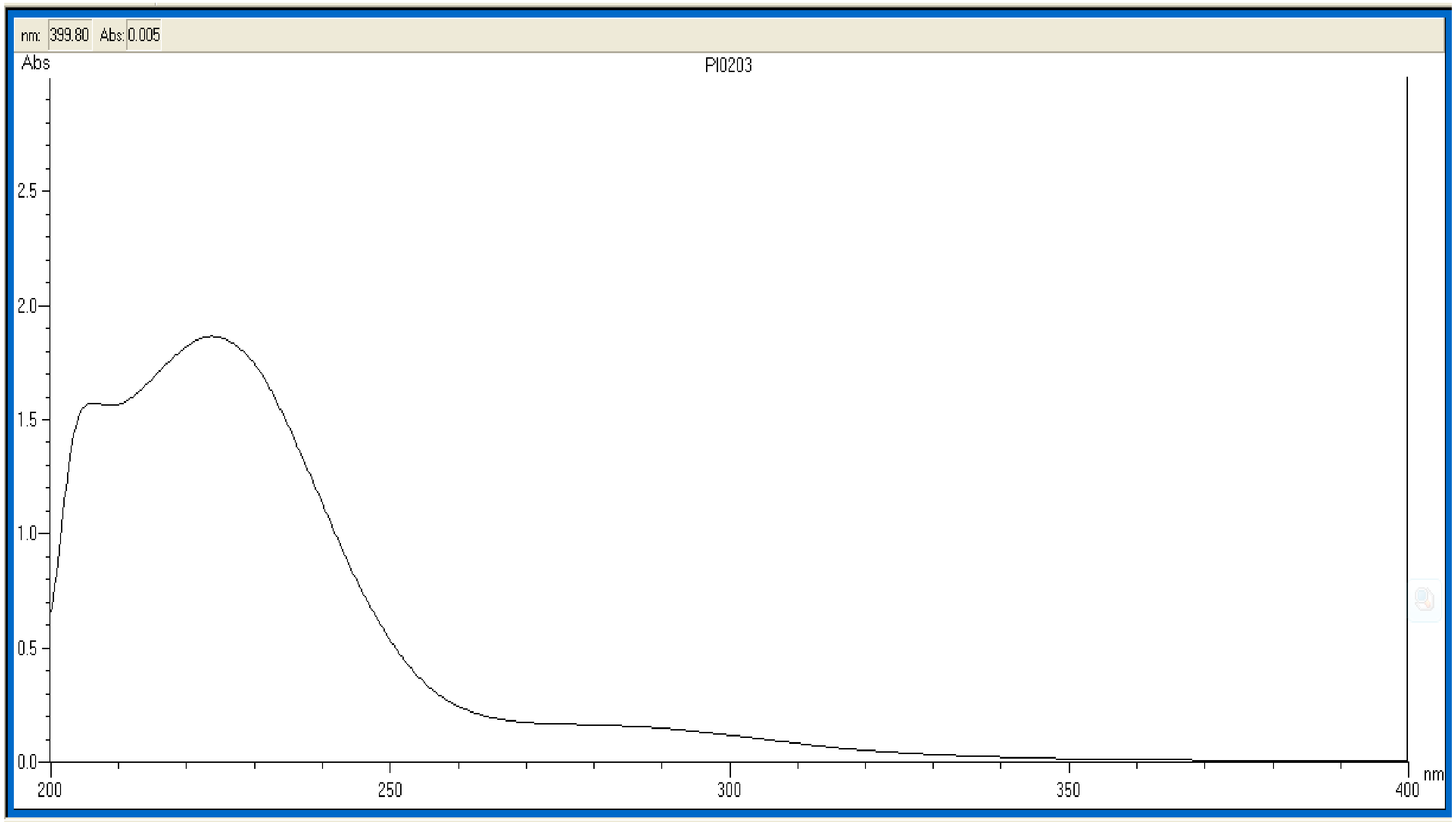

PI0203

Peak #	Start (nm)	Apex (nm)	End (nm)	Height (Abs)	Valley (nm)	Valley (Abs)
1	400.00	223.60	208.80	1.865	208.80	1.563
2	208.80	205.80	200.00	1.574	200.00	0.654

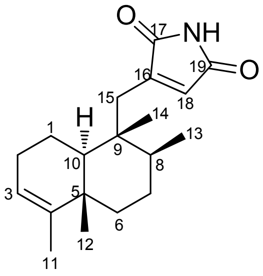

Dysidinoid A (**1**)

Figure S10. IR spectrum of Dysidinoid A (**1**).

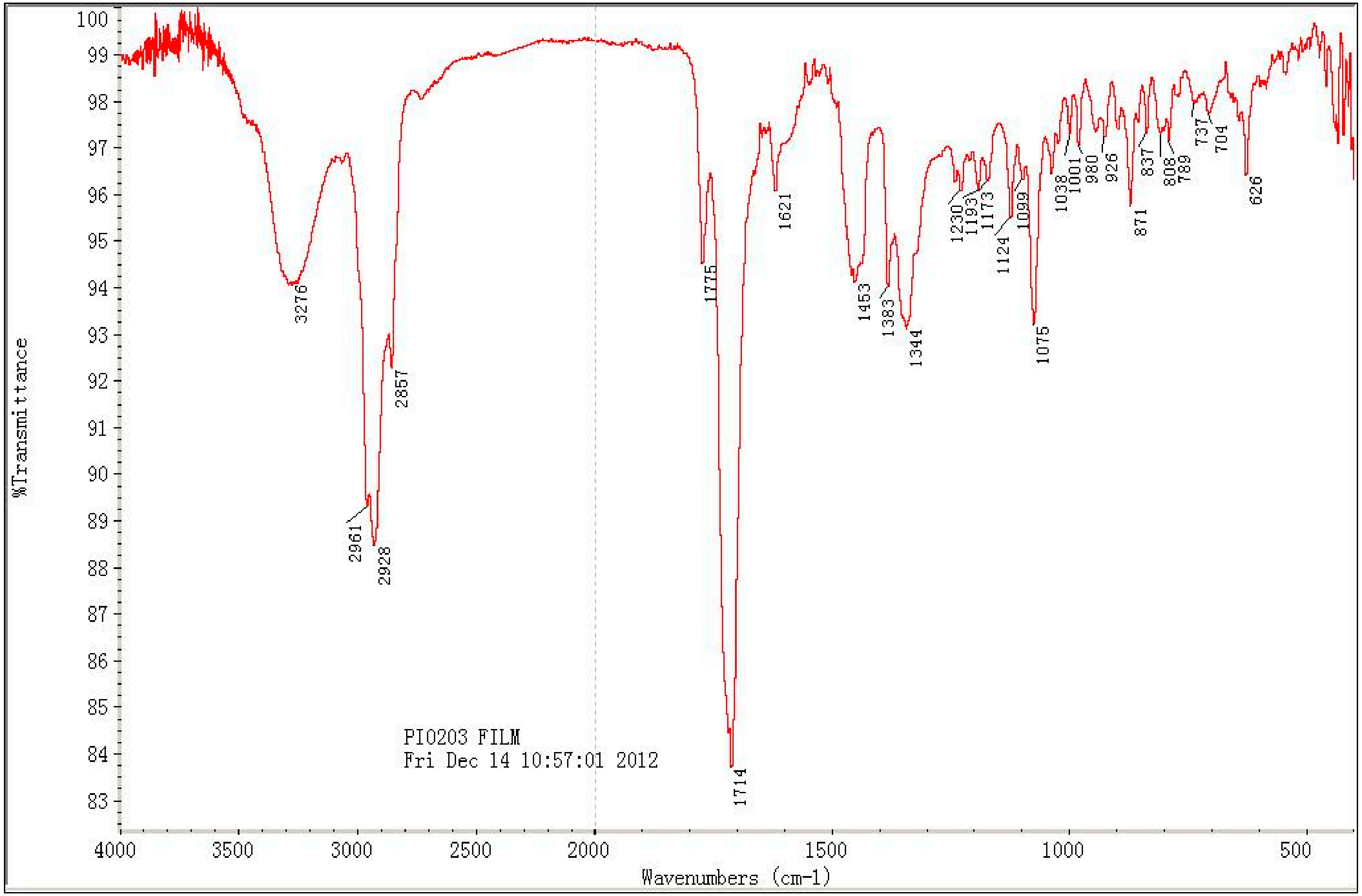

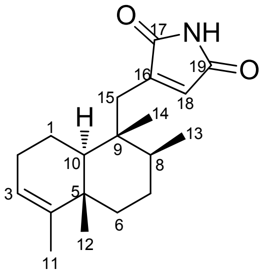

Dysidinoid A (**1**)
